# How to deal with context? A context-mapping tool for quality and safety in nursing homes and homecare (SAFE-LEAD Context)

**DOI:** 10.1186/s13104-019-4291-3

**Published:** 2019-05-10

**Authors:** Siri Wiig, Karina Aase, Terese Johannessen, Elisabeth Holen-Rabbersvik, Line Hurup Thomsen, Hester van de Bovenkamp, Roland Bal, Eline Ree

**Affiliations:** 1 SHARE-Centre for Resilience in Healthcare, Faculty of Health Sciences, University of Stavanger, Stavanger, Norway; 2 Department of Health and Nursing Sciences, University of Agder, Songdalen Municipality, Kristiansand, Norway; 3 Center for Developing Institutional and Home Care Services Rogaland, Stavanger municipality, Stavanger, Norway; 4 Erasmus University, School of Health Policy & Management, Rotterdam, The Netherlands

**Keywords:** Context mapping, Quality improvement, Patient safety, Nursing home, Homecare, Cross-country comparison

## Abstract

**Objective:**

The objective of this paper is to develop a context-mapping tool (SAFE-LEAD Context) adapted to the nursing home and homecare setting. These two contexts represent a substantial variability, but studies focusing on the types and roles of contextual factors in quality and safety in these care settings are lacking.

**Results:**

We conducted a step-wise collaborative design process consisting of mapping of key contextual factors as perceived by managers in Norwegian nursing homes and homecare, then created a draft tool discussed in a consortium workshop with co-researchers, and ran an international cross-country comparison. The SAFE-LEAD Context tool is inspired by the Consolidated Framework for Implementation Research (CFIR). The tool incorporates factors describing the outer setting of nursing homes and homecare at the national and local levels, in addition to factors describing the inner setting. The tool is flexible yet more detailed than current frameworks and capable of grading and describing the included contextual factors over time in the nursing home and homecare settings. A systematic approach using the SAFE-LEAD Context tool will support and improve the understanding and evaluation of quality and safety improvement interventions.

## Introduction

There is a dearth of literature about what kind of and how contextual factors influence knowledge translation [1–4] and the continuous quality and safety work in healthcare services [5–9]. Context can be conceptualized as a set of circumstances or factors that surround improvement efforts [10], and can refer to both the inner (internal) and outer (external) settings of an organization. Internal organizational factors include structural characteristics (e.g., location and size), the local working environments of teams and leadership, and the organizational culture and implementation climate. External factors include applicable laws, regulatory requirements, external policies and incentives, funding structures [8], patient organizations, payers, and professional organizations [11]. Context is not independent of the actors within specific healthcare settings; rather, it is something that can be acted upon and changed [12].

In the international body of literature, most of the research on improving quality and safety in healthcare is conducted in the hospital setting so we know less about other settings [13]. Health services provided by nursing homes and homecare are essential in most countries, and the quality and safety work in these settings is attracting increased attention [14]. The different settings that nursing home and homecare services operate within vary greatly, and there are few studies of the types and roles of contextual factors in these care settings [15–17]. Therefore, the objective of this paper is to develop a context-mapping tool (SAFE-LEAD Context) that is tailored to the nursing home and homecare settings.

Our SAFE-LEAD Context tool was developed as part of the SAFE-LEAD project [18]. It is inspired by McDonald’s [8] operationalization of the Consolidated Framework for Implementation Research (CFIR) [10]. The CFIR focuses on implementation research and consists of five domains (1) intervention characteristics; (2) outer setting; (3) inner setting; (4) characteristics of the individuals involved; and (5) implementation. We extended, developed, and adapted the domains of inner and outer settings to the nursing home and homecare settings. In the CFIR framework [10], outer setting consists of:

- Patient needs and resources
- Cosmopolitanism (extent of external network)
- Peer pressure (to implement interventions)
- External policies and incentives

The inner setting refers to:

- Structural characteristics
- Networks and communication
- Culture
- Implementation climate and readiness

## Main text

### Results

#### Development setting

In Norway, municipalities are by law responsible for providing nursing home and homecare services to residents, and the managers have a clearly defined role in ensuring service quality and safety [19, 20]. The requirements for quality and safety are the same across all municipalities, although size, geographical location, and competence varies greatly from large cities to small rural areas.

#### A step-wise collaborative design process

**Fig. 1:**
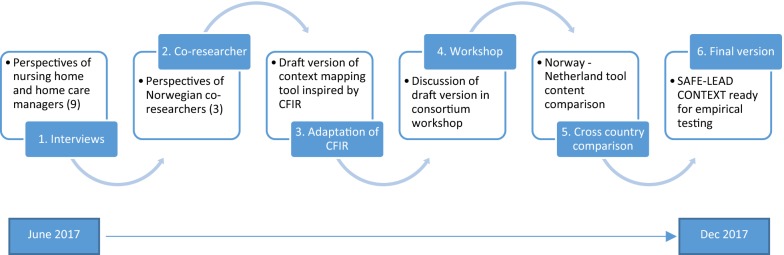
Methodological design steps in the SAFE-LEAD Context development

In a collaborative development process, we applied the design steps depicted in Fig. 1, which were: (1) qualitative interviews with managers in nursing homes and homecare in Norway; (2) input from co-researchers with broad experience from the Norwegian nursing homes and homecare services; (3) assessment of CFIR and drafting of the tool; (4) workshops with researchers, user representatives and practice-based co-researchers; (5) iterative cross-country comparison of tool contents; and (6) finalizing the SAFE-LEAD Context tool.

Step 1 consisted of a qualitative interview study with nine nursing home and homecare managers in six Norwegian municipalities (large, small, rural, city). The participants were top and middle managers within those municipalities, and represented different regions, geographical locations and institutions of different sizes. All of these managers were educated as registered nurses and had experience as frontline staff. The participants were purposely selected to maximize their contextual diversity. Residents and frontline staff were not included in step 1 as the focus was managers’ experience with working on quality and safety. Three practice-based co-researchers in the SAFE-LEAD project consortium working in different Norwegian regions recruited the participants. The interview guide included open questions regarding which factors managers perceived as important for their work with quality and safety, and topics such as external factors, economy, and structure. Each interview lasted approximately 45 min and was audiotaped. All interviews were transcribed and subjected to thematic analysis [21]. For all themes, different contextual factors were noted to specify potential topics or questions to be included in the context-mapping tool (see Table 1). This was discussed in an analysis workshop attended by SW, ER, TJ, LHT, EHR, and additional project members.

**Table 1:** Summary of themes and contextual factors identified by managers (step 1)

Themes	Contextual factors
Geographical location, municipality size	Size of municipality Geographical distance to hospital/within the municipality Access to proper competence and networks in the municipality Number of departments/organizational size/employees
Access to resources and proper competence	Financial situation/time pressure Access to doctors and nurses (recruitment) Existing resource groups/persons/professional development positions Competence in the organization
Organizing of services, distribution of responsibility	Organizing of quality and safety Managerial levels Type of services and user/patient groups Treatment level Team organizing Communication with decision makers in the municipality
Systems and tools for QI	Type of incident reporting system (paper-based/digital) Use of checklists Use of register or monitoring system for patient indicators
Network within and outside the municipality to support managers	Committees (quality, patient safety, user) Research and development unit Contact with development centers in the region Employed doctor at the nursing home Resource groups at the municipal/regional/national level
External demands and guidelines	Key national policy documents and regulation Demands for documentation Participation in national programmes Care coordination demands and safety in transitional care
Communication, culture, and meeting points as part of the managerial work	Meeting arenas between managers and healthcare professionals Meeting arenas for managers Functionality of IT-systems as communication tool
User involvement in user-panels, user surveys	User panel Elderly user panel/next-of-kin panel/next-of-kin representation in user panel Use of user surveys
Current change processes within the municipality	Ongoing/recent organizational change processes Resistance to change Current implementation of improvement measures

In step 2, we asked the three co-researchers to provide written notes (complementing the thematic analysis), on what they considered the ten most important contextual factors, based on their diverse background and experience as managers and healthcare professionals (nurses) in primary care (nursing home, homecare, development center for institution and homecare services).

In step 3, based on the factors identified from the thematic analysis, the written notes from the co-researchers, and assessment of the CFIR, we assessed what additional factors that should be included to cover the nursing home and homecare settings. SW, ER, EHR, and TJ drafted a first version of the context-mapping tool.

In step 4, we conducted a context-mapping design workshop with all the Norwegian consortium partners and co-researchers to obtain feedback on the draft version. In this workshop, user representatives including one senior representative and one Patient and user ombudsman participated. Both are members of the project consortium with in depth knowledge of the nursing home and homecare settings. Here we discussed the dimensions going into the tool, how data could be collected to map the factors over time, and whether those factors could be assessed on a five-point scale.

In step 5, we conducted an iterative cross-country comparison of tool contents with the Dutch researchers RB and HvB in the consortium, who assessed its relevance from an international perspective and suggested additional factors. The Dutch researchers focused on whether the tool included relevant contextual factors to enable a cross-country comparison of quality and safety work and interventions. Step 5 was supported by a review of macro-level factors for understanding quality and safety improvement efforts across countries [22].

In step 6, we finalized the SAFE-LEAD Context tool and prepared it for empirical testing.

#### Key contextual factors

Based on the analysis of the nine manager interviews (step 1), Table 1 depicts the identified common themes and specific contextual factors. The main issues were related to the size of the municipality, the size of the nursing home or homecare service provider, and geographical location. Organizations were sometimes considered too large or small, with long driving distances to a hospital or to the service users in the rural areas, and this made a manager’s work on improving quality and safety more complicated. Other factors that appeared important pertained to care coordination, collaboration, and relation to the elected politicians in the municipality. Budget constraints, difficulties with collaboration and coordination across service levels were noted as challenges in daily operations. External demands in terms of regulation, national guidelines, and national policy documents both supported and hindered the local improvement work. Policy documents pinpointing the role of managers’ responsibility for improving care quality and safety, supported their effort and contributed to put the topic on the agenda both within the nursing home or homecare organizations, and at the municipal level. At the same time, the external demands could be overwhelming due to resource constraints and limited competence. Access to relevant competence and capacity varied across the municipalities and recruitment could be especially difficult in rural areas. Access to resources (time and money) was furthermore focused, and there was a consensus that chronic lack of time and increasing demands for efficiency hampered managers’ ability to devote sustained attention to the improvement of quality and safety.

The structural aspects related to status of IT systems, incident reporting systems, checklists, and documentation varied among the participants. Many emphasized the importance of incident reporting systems, but there was a range of IT systems and access to computers among healthcare staff. The managers also considered cultural factors and leadership as key themes for the work on quality and safety. They acknowledged their responsibility as role models and the importance of building an understanding for the need for improving quality and safety in tandem with the team of healthcare professionals.

In step 2 of our design process, the practice-based co-researchers confirmed the contextual factors summarized in Table 1, focusing on the following factors:

- Collaboration and relations: (a) between local politicians and managers; (b) among different healthcare professionals (nurses, doctors, physiotherapists, and occupational therapists); and (c) with research institutions
- Dedicated resources to quality and safety (competence, time, personnel)
- Continuity of care within and across service levels
- Nurse-patient ratio
- Location, travel distance to hospital
- Digital infrastructure

#### The context-mapping tool (SAFE-LEAD Context)

Table 2 presents the final version of the SAFE-LEAD Context tool for identifying contextual factors for quality and safety in nursing homes and homecare. The tool includes factors describing the outer setting at the national and local levels, in addition to factors describing the inner setting. The tool opens for grading and describing the included contextual factors over time using a scale from 1 (low degree/small) to 5 (high degree/large). We added the grading possibility to enable descriptive comparison between different units involved in the mapping or to track potential change over time. This possible specification of degree adds to the original CFIR, which mentions “the degree of which” for several constructs but does not include any specific grading. For some factors, this grading is not applicable, and we therefore added a column for free text assessment and/or description of the factor.

**Table 2:** The SAFE-LEAD Context tool adapted from CFIR

Context domain	Domain description	Assessment/description	Grade 1–5 (1 = low/small 5 = high/large)				
			1	2	3	4	5
Outer setting	(Outside municipality—national level)						
External policy and incentives	National strategies to spread interventions						
	National program for quality and safety						
	Degree of national support for quality and safety work/competence						
	Degree of available national quality indicators						
	Degree of national digital quality and safety tools						
Regulatory framework	Enforced self-regulation/control/accreditation/insurance						
	Degree of regulatory pressure on managers						
	Supervisory authority for quality of care						
Role of state in organizing of nursing homes and homecare	Delegated to municipalities by law/state run/other						
Funding	Degree of use co-payment of services						

Context domain	Domain description	Assessment/description	Grade 1–5 (1 = low/small 5 = high/large)				
			1	2	3	4	5
Outer setting	(Within municipality—local level)						
Patient needs and resources	The extent to which patient needs are known and prioritized						
Citizen involvement	Degree of citizen involvement in the municipality						
Cosmopolitanism	Degree organization is networked with other external organizations						
	Degree of collaboration between municipalities in quality and safety						
	Degree of local support and competence for quality and safety						
External policy and incentives	External strategies to spread interventions						
Municipality size, location	Number of inhabitants/city, rural						
Distance to hospital	Hours to drive from nursing home/homecare						
Type of funding	Private/public						
Digital infrastructure	Degree of development of digital infrastructure including electronic error reporting systems						
Collaboration climate	Degree collaboration between politicians and managers						
Financial status	Degree of financial pressure to save costs						

Context domain	Domain description	Assessment/description	Grade 1–5 (1 = low/smal 5 = high/large)				
			1	2	3	4	5
Inner setting	(Within institution—organizational level)						
Type of service	Homecare/nursing home/level of treatment (describe)						
Structural characteristics	Social architecture—degree of how many employees are clustered into smaller groups						
	Nurse-patient ratio						
	Number of managerial levels within institution						
	Assessment of manager-employee ratio						
	Institution size						
	Degree of quality/safety infrastructure						
External demands	Degree of consistency between external demands and clinical practice						
Patient and user involvement in quality and safety improvement	Degree of possibilities for involvement of user/patient/next of kin involvement at system level (arenas, board, committees, survey, co-design)						
	Degree of actual involvement of user/patient/next of kin at system level (arenas, board, committees, survey, co-design)						
Patient/user centeredness	Degree of user/patient centeredness in service provision						
Work schedule	Degree of organizing of work schedule according to patient needs						
Workforce	Degree of age, maturity among staff						
	Degree of part-time employment						
	Degree of doctor availability						
	Degree of nurses availability						
	Degree of unskilled employees in the work force						
	Degree of access to, and use of, inter-professional competence such as psychologist, occupational therapist and physical therapist						
Competence	Degree of competence level among work force (registered nurses, resource groups, improvement team, professional development nurses)						
	Degree of delegating responsibility in acquiring knowledge in specific subjects to staff						
Engagement	The degree managers support and engage staff in quality and safety improvement work						
Networks and communications	Nature and quality of social networks, formal and informal communication						
	Degree of arenas and structure for inter-professional collaboration						
	Degree of attention to handover as a risk area						
Culture	Norms, values, and basic assumptions of organization						
	Degree of interest in improvement work within the organization						
Implementation climate	Capacity for change, shared receptivity to improvement interventions, extent to which use improvement is rewarded, supported, and expected within organization						
Readiness for implementation	Tangible indicators of organizational commitment to quality and safety improvement intervention						
Availability of resources	Degree of available time to work with improvement						
	Degree of available funding for improvement work						
Autonomy	Degree of autonomy in how to utilize available resources						

### Discussion

The success of quality and safety efforts depends on contextual factors [23–26]. Most research on the topic has been conducted in hospitals so less is known about the role of contextual factors in nursing homes and homecare. In this paper, we have demonstrated our step-wise collaborative design process in developing a context-mapping tool. We mapped key contextual factors as perceived by managers, co-researchers, user representatives, international researchers, and developed SAFE-LEAD Context, inspired by the CFIR, to support understanding and evaluation of improvement efforts in the nursing home and homecare settings. The CFIR framework was chosen as a basis for the SAFE-LEAD Context tool, as it provides a list of constructs and argues that each construct should be carefully reviewed and fitted to the setting at hand [10]. The SAFE-LEAD Context tool supports targeted context factor mapping in nursing homes and homecare, in a Norwegian and international perspective. We are confident that other researchers or practitioners can apply the tool or replicate its development. We argue that using a similar collaborative development approach, including user-representatives and co-researchers, when adapting the CFIR or other frameworks, will support knowledge translation or intervention studies to improve quality and safety in their specific setting.

The SAFE-LEAD Context tool is currently being tested in an intervention study including four nursing homes and four homecare services in Norway [18], and results including the evaluation of the tool will be published as part of the project publication plan.

## Limitations

- This paper describes the development process of the SAFE-LEAD Context tool. This version has not yet been empirically tested for effectiveness and applicability
- The sample of managers and practice-based co-researchers is limited and should be expanded to additional primary care settings
- The sample could have included frontline staff, patients, and users
- The international cross-country component should be expanded beyond researchers to managers and user-representatives from different countries

## Data Availability

The datasets used and/or analysed during the current study are available from the corresponding author on request.

## Abbreviation

CFIR: Consolidated Framework for Implementation Research
